# Sinus- and Sequence-Specific Diagnostic Performance of Routine Unenhanced Brain MRI in Dural Venous Sinus Thrombosis

**DOI:** 10.3390/diagnostics16121771

**Published:** 2026-06-08

**Authors:** Mehmet Karagulle, Tahsin Benlice, Tuba Banaz, Burak Kocak

**Affiliations:** Department of Radiology, Basaksehir Cam and Sakura City Hospital, Istanbul 34480, Türkiye; tbenlice@gmail.com (T.B.); tubabanaz59@gmail.com (T.B.); drburakkocak@gmail.com (B.K.)

**Keywords:** dural venous sinus thrombosis, magnetic resonance imaging, MR venography, inter-reader agreement, cerebral venous thrombosis

## Abstract

**Background:** Dural venous sinus thrombosis (DVST) is an uncommon but potentially life-threatening cerebrovascular disorder requiring early diagnosis to prevent serious complications. Although CE-MRV is the reference standard, routine brain MRI is often the first imaging study in patients with nonspecific neurological symptoms, and the sinus-specific diagnostic performance of individual sequences remains incompletely defined. **Purpose:** To evaluate the diagnostic performance and inter-reader agreement of routine brain MRI sequences for DVST detection using a sinus-specific framework. **Methods:** This retrospective case–control study included 140 patients (34 with DVST, 106 age-matched controls) imaged on 1.5 T and 3.0 T scanners. Two blinded neuroradiologists evaluated six unenhanced sequences (sagittal/axial T1WI, T2WI, FLAIR, DWI [b = 1000 s/mm^2^], and SWI) across four dural sinuses, using CE-MRV and CE-3D T1WI as the reference standards. Logistic regression and Cohen’s κ assessed diagnostic performance and inter-reader agreement, respectively. **Results:** Globally, DWI with FLAIR achieved 97.9% accuracy, 91.2% sensitivity, and 100% specificity (AUC = 0.997). Optimal sequences varied by sinus: sagittal T1WI with SWI for the superior sagittal sinus (accuracy = 99.3%), DWI with SWI for the transverse sinus (97.9%), DWI with FLAIR and T2WI for the sigmoid sinus (98.6%), and SWI with axial T1 for the straight sinus (100%). Inter-reader agreement was substantial to almost perfect for routine sequences (mean κ = 0.874) and almost perfect for CE-MRV and CE-3D T1WI (κ = 0.98). **Conclusions:** Routine brain MRI provides reliable DVST detection with a sinus-tailored multisequence strategy. DWI and FLAIR offer robust diagnostic performance in global evaluation, while T1WI, SWI and T2WI add segment-specific value, reserving CE-MRV and CE-3D T1WI for equivocal or clinically suspicious cases.

## 1. Introduction

Dural venous sinus thrombosis (DVST) is a rare but potentially life-threatening cerebrovascular disorder with an annual incidence of 1–2 per 100,000 individuals [1]. If untreated, DVST can lead to venous infarction, hemorrhagic transformation, or intracranial hypertension, resulting in significant morbidity and mortality [1,2]. Clinical manifestations range from isolated headache to coma, contributing to frequent underrecognition, particularly in younger patients or those lacking classical predisposing factors such as prothrombotic disorders, infections, trauma, or inflammatory conditions [1,3].

Early diagnosis is critical, as prompt anticoagulation substantially reduces morbidity and mortality [3,4]. However, diagnostic delay remains common because patients frequently present with nonspecific neurological symptoms and undergo routine brain magnetic resonance imaging (MRI) as the initial imaging modality before dedicated vascular imaging is considered [5,6]. Although contrast-enhanced magnetic resonance venography (CE-MRV) is the established reference standard, it is often not part of the initial emergency protocol [3,5].

Routine unenhanced brain MRI may reveal direct signs of DVST, including T1-weighted imaging (T1WI) hyperintensity within the sinus, absence of the normal flow void on T2-weighted imaging (T2WI), restricted diffusion on diffusion-weighted imaging (DWI), and susceptibility effects on susceptibility-weighted imaging (SWI) [7,8,9]. However, the diagnostic reliability of these findings varies considerably across sequences and individual dural sinuses. Misinterpretation of these subtle signs remains a clinical challenge; false-positive assessments may lead to unnecessary further imaging and anticoagulation, while false-negative results can dangerously delay life-saving treatment [3,10,11,12].

Prior studies have largely evaluated individual sequences in isolation or focused on specific sinuses, without applying a comprehensive, sinus-specific analytical framework across the full range of routine MRI sequences [7,8,9,10]. A better understanding of sequence- and sinus-dependent diagnostic performance is essential to improve interpretation accuracy and guide more efficient imaging strategies.

Therefore, this study aimed to evaluate the diagnostic performance and inter-reader agreement of routine unenhanced brain MRI sequences for DVST detection, using CE-MRV and contrast-enhanced 3D T1-weighted imaging (CE-3D T1WI) as reference standards. A sinus-specific analytical framework was applied to identify the most diagnostically informative sequence or sequence combination for each major dural venous sinus.

## 2. Materials and Methods

### 2.1. Ethical Approval

This retrospective study was approved by the Local Ethics Committee of Başakşehir Çam and Sakura City Hospital (Approval No: 2025-02; 15 January 2025), with waived written consent. The study followed the Declaration of Helsinki.

### 2.2. Study Population

A retrospective search of radiology reports from 2021–2025 was conducted using the following keywords: “dural venous sinus thrombosis,” “venous sinus thrombosis,” and “sinus thrombosis,” identifying patients with available MRI scans. Only patients with a complete MRI protocol—including CE-MRV, CE-3D T1WI, SWI, DWI (b = 1000 s/mm^2^), sagittal T1WI, and axial FLAIR, T1-, and T2-weighted imaging—were eligible.

Patients were excluded if they had an incomplete MRI protocol, motion or susceptibility artifacts precluding diagnostic evaluation, tumor invasion of the dural venous sinus, atretic venous sinus, or a history of prior sinus surgery.

Eligible examinations were re-evaluated for DVST by a neuroradiologist with 7–8 years of experience, blinded to clinical data and original reports, using CE-MRV and CE-3D T1WI as reference standards. Among DVST-negative patients, controls were selected using a computer-generated random sampling algorithm with frequency matching for age. To optimize statistical power while preserving group comparability, an approximate 1:3 case-to-control ratio was used, as increasing the number of controls beyond this threshold yields diminishing returns in statistical efficiency [13].

### 2.3. Imaging Protocol

MRI examinations were performed on three MRI systems at two field strengths: two 1.5-Tesla scanners (Ingenia, Philips Healthcare, Best, The Netherlands; Echelon smart, Fuji, Tokyo, Japan) equipped with 16-channel head coils, and a 3.0-Tesla scanner (Ingenia, Philips Healthcare, Best, The Netherlands) equipped with a 32-channel phased-array head coil.

Contrast enhancement was achieved via automated power injection (Spectris MR Injector; MedRad, Warrendale, PA, USA) of various gadolinium-based contrast agents at a dose of 0.1 mmol/kg (maximum 10 mL) and an injection rate of 2 mL/s.

Contrast-enhanced 3D T1-weighted imaging was acquired using gradient-echo-based sequences, including Turbo Field Echo (TFE) on Philips systems and RSSG on Fuji systems. Susceptibility-weighted imaging (SWI) was performed using a single-echo sequence on the 1.5 T Fuji system, whereas a multi-echo fast field echo (mFFE) sequence was used on Philips systems. Pre-contrast sagittal T1-weighted imaging was obtained using Turbo Spin Echo (TSE) on Fuji systems and TFE on Philips systems. Pre-contrast axial T1-weighted imaging was performed using TSE on 1.5 T Fuji and Philips systems, while 3 T Philips systems used TFE. Additionally, FLAIR sequences were acquired as 3D FLAIR on Philips systems. Detailed representative imaging parameters are provided in Table 1.

### 2.4. MRI Signs Evaluated for DVST

Each sinus was evaluated against adjacent normal segments using these criteria:

- **Sagittal and Axial T1WI:** Intraluminal hyperintensity.
- **Axial T2WI:** Absent flow void or intraluminal hyperintensity.
- **FLAIR:** Absent flow void or intraluminal hyperintensity.
- **Axial SWI:** Marked hypointensity with blooming artifact.
- **Axial DWI (b = 1000 s/mm^2^):** Intraluminal diffusion restriction.
- **CE-3D T1WI and CE-MRV:** Intraluminal filling defect.

### 2.5. Image Analyses

Images were anonymized and organized into two datasets: (1) unenhanced sequences (sagittal T1WI, axial T1WI, T2WI, FLAIR, SWI, and DWI) and (2) contrast-enhanced sequences (CE-3D T1WI and CE-MRV).

Two neuroradiologists with 5 years of experience, blinded to clinical data, radiology reports, and each other’s assessments, independently evaluated the unenhanced dataset, assessing superior sagittal, straight, transverse, and sigmoid sinuses. The same readers subsequently evaluated the contrast-enhanced dataset in a separate session at least one week later, remaining blinded to prior assessments. Consensus decisions of these two readers were used in diagnostic accuracy evaluations against the reference standard (defined by the more experienced neuroradiologist). Patients were classified as DVST-positive if thrombosis was detected in at least one sinus segment.

### 2.6. Statistical Analysis

Group differences in age were evaluated using the Mann–Whitney U test and sex–DVST associations using the chi-square test. Diagnostic associations were analyzed using binary logistic regression at two levels: global sequence-based and sinus-specific. Model fit was assessed using pseudo-R^2^ indices; because several predictors exhibited quasi-complete separation, interpretation emphasized model-level performance metrics from confusion matrices. Inter-reader agreement was assessed using Cohen’s kappa. All analyses were conducted in JASP (version 0.95.4; JASP Team, University of Amsterdam, Amsterdam, Netherlands; Apple Silicon), and Jamovi (version 2.6.45.0; Sydney, Australia; Apple Silicon), with significance set at *p* < 0.05.

## 3. Results

### 3.1. Study Population and Cohort Formation

**Figure 1:**
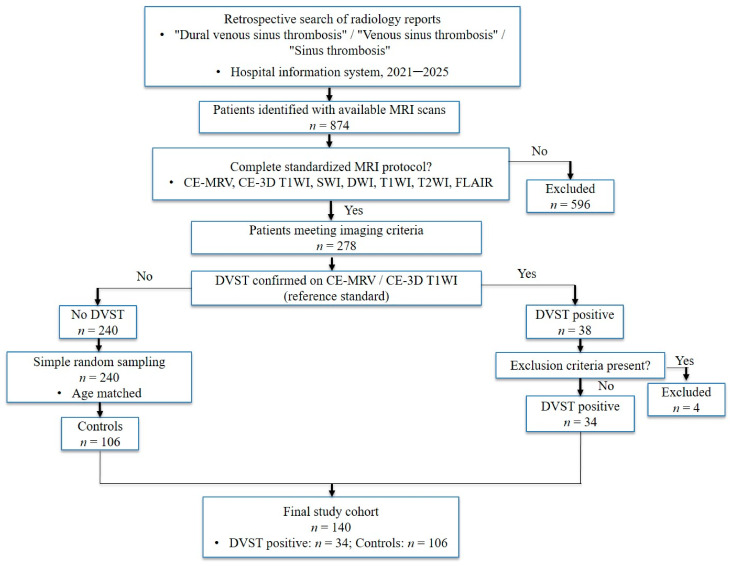
Study flow chart.

Of the 874 patients identified, 596 were excluded: 462 lacked reference sequences, 103 were missing one or more required sequences, and 31 had motion or susceptibility artifacts. The remaining 278 scans were re-evaluated for DVST by a neuroradiologist with 7–8 years of experience, blinded to clinical data and reports, using CE-MRV and CE-3D T1WI as reference standards. DVST was confirmed in 38 patients; four were excluded due to tumor invasion, atretic sinus, or prior surgery. Among the 240 DVST-negative patients, 106 controls were selected using a computer-generated random sampling algorithm with frequency matching for age. The final study cohort comprised 140 subjects (34 cases and 106 controls; Figure 1).

### 3.2. Patient Characteristics

Of the 140 included patients, 34 (24.3%) had DVST and 106 (75.7%) did not. Age did not differ significantly between groups (mean 31.5 vs. 27.6 years; mean ranks 72.1 vs. 70.0; U = 1747.5, *p* = 0.793), and the effect size was negligible (rank-biserial correlation = 0.03). Sex distribution differed between groups (χ^2^ = 5.60, *p* = 0.018), with a higher thrombosis proportion in males (34.5%) than in females (17.1%). The most common etiologies among DVST cases were otomastoiditis and idiopathic thrombosis (*n* = 10 each), followed by hematologic malignancies (*n* = 3), Behçet’s disease (*n* = 3), and tamoxifen use (*n* = 3). Other identified causes included antiphospholipid syndrome, hypereosinophilic syndrome, oral contraceptive use, trauma, and lung carcinoma (all *n* = 1). The majority of subjects were imaged on the 1.5-Tesla system, including 30 of 34 cases (88.2%) and 92 of 106 controls (86.8%); the field-strength distribution was comparable between groups (Fisher’s exact test, *p* = 1.000).

### 3.3. Global Sequence-Based Analysis

In the global sequence-based analysis (Table 2), the combined model incorporating restricted diffusion on DWI b1000 and axial FLAIR hyperintensity showed excellent fit (McFadden R^2^ = 0.917) and almost perfect discrimination (AUC = 0.997), achieving 97.9% accuracy, 91.2% sensitivity, and 100% specificity with no false-positive predictions.

A positive finding on any conventional MRI sequence (T1WI, T2WI, FLAIR, DWI, or SWI) served as a highly sensitive initial indicator of thrombosis (100% sensitivity; 93.6% accuracy), although with a greater number of false-positive classifications compared with DWI/FLAIR.

Across all global models, extremely large coefficients with inflated standard errors indicated quasi-complete separation, consistent with imaging abnormalities that almost perfectly distinguished thrombosed from non-thrombosed cases; therefore, diagnostic accuracy metrics were prioritized.

### 3.4. Sinus-Specific Analysis

In the sinus-specific analysis (Table 3), diagnostic performance remained consistently high, with the dominant predictor varying by dural venous sinus segment.

For the superior sagittal sinus (SSS), T1WI sagittal hyperintensity was the strongest marker (McFadden R^2^ = 0.794), with SWI providing additional discriminatory value (final McFadden R^2^ = 0.883). Accuracy reached 99.3%, with 100% specificity and 93.8% sensitivity.

Regarding the transverse sinus, DWI b1000 hyperintensity strongly predicted thrombosis (McFadden R^2^ = 0.797), and adding SWI enhanced the model (McFadden R^2^ = 0.939). The combined model achieved 97.9% accuracy, AUC 0.999, 100% sensitivity, and 97.3% specificity.

For the sigmoid sinus, DWI b1000 was again the dominant predictor (McFadden R^2^ = 0.746), with incremental contribution from FLAIR and T2-weighted hyperintensities (final McFadden R^2^ = 0.881). Performance remained excellent (98.6% accuracy, AUC 0.980, 92.6% sensitivity, 100% specificity).

Regarding the straight sinus, SWI and axial T1WI hyperintensity together provided perfect discrimination (100% accuracy, AUC 1.000, 100% sensitivity and specificity) with no misclassifications.

**Figure 2:**
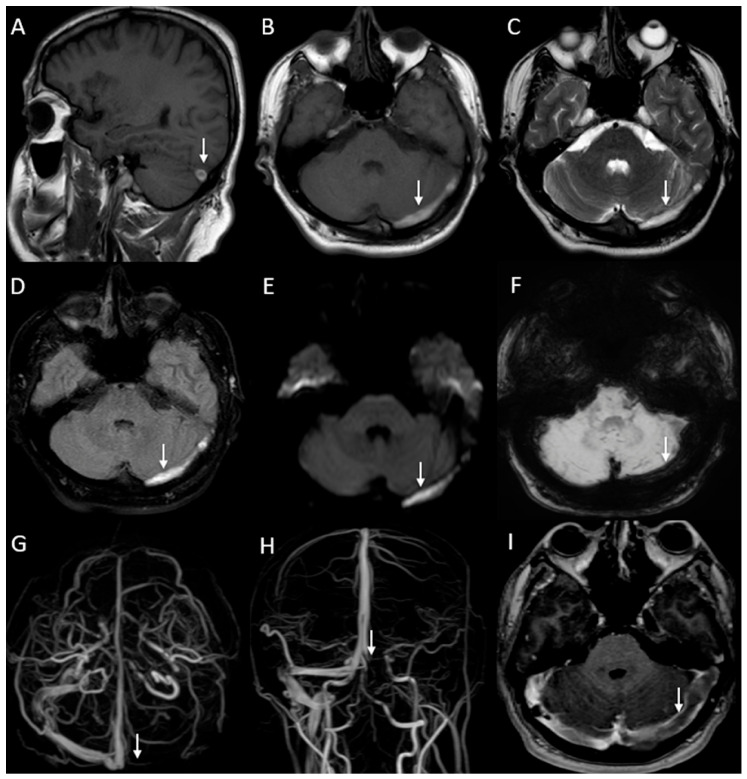
MR imaging signs of dural venous sinus thrombosis in a representative case. (**A**), Hyperintense signal in the left transverse sinus on sagittal T1WI. (**B**), Hyperintense signal in the left transverse sinus on axial T1WI. (**C**), Loss of flow void in the left transverse sinus on axial T2WI. (**D**), Hyperintense signal in the left transverse sinus on FLAIR. (**E**), Hyperintense signal in the left transverse sinus on DWI. (**F**), Blooming artifacts in the left transverse sinus on SWI. (**G**,**H**), Filling defect in the left transverse/sigmoid sinus on MIP-CE-MRV. (**I**), Filling defect in the left transverse/sigmoid sinus on CE-3D T1WI. The thrombosed sinus is indicated by white arrows in all images.

Across all analyses, a small subset of MRI sequences consistently demonstrated the highest diagnostic value. DWI b1000 was the strongest overall sequence, providing outstanding discrimination in the transverse and sigmoid sinuses. T1WI was the key discriminator in the SSS, while SWI was highly reliable across regions and achieved perfect classification in the straight sinus when combined with axial T1WI hyperintensity. FLAIR contributed meaningfully to the sigmoid sinus evaluation but was not a dominant standalone predictor. Overall, DWI, T1WI, and SWI emerged as the principal sequences providing the highest diagnostic performance. Representative multisequence MRI findings in a confirmed DVST case, including intraluminal T1 hyperintensity, DWI restriction, and SWI blooming artifacts, are illustrated in Figure 2.

### 3.5. Reliability Analysis

Inter-reader agreement was excellent in global sequence-based analysis (Table 4). Cohen’s kappa values for the six conventional sequences (T1WI sagittal, T1WI axial, T2WI axial, FLAIR axial, DWI b1000, and SWI) ranged from 0.821 to 0.898, with a mean kappa of 0.874, indicating substantial-to-almost-perfect agreement. Agreement for contrast-based assessments was almost perfect, with a kappa value of 0.98 for both CE-MRV and CE-3D T1WI sequences.

Inter-reader agreement for all sinus-level ratings was high. Across transverse, sigmoid, and SSS, most sequences demonstrated substantial-to-almost-perfect reliability, with kappa values generally above 0.75, and CE-MRV and CE-3D T1WI sequences consistently reached the highest agreement. The straight sinus showed perfect concordance for several sequences, with lower kappa values limited to axial T1WI and T2WI sequences. Table 5 demonstrates reliability across all venous sinuses.

## 4. Discussion

This study evaluated the diagnostic performance of routine brain MRI sequences for detecting DVST using a sinus-specific approach with validation against contrast-enhanced reference standards. Overall, routine MRI demonstrated high diagnostic performance, particularly when multiple sequences were interpreted together [7,14]. The combination of DWI and FLAIR provided the most balanced global performance, offering very high specificity and strong sensitivity, although not all cases are detected with this combination alone. SWI further improved thrombus visualization, particularly in the straightand transverse sinuses, where it offered a more objective assessment than conventional flow-void evaluation. T1WI was most accurate in the SSS using sagittal acquisitions. The transverse sinus was the most diagnostically challenging segment, showing a higher rate of false-positive findings and lower inter-reader agreement on axial T2WI. Contrast-enhanced sequences demonstrated almost perfect inter-rater agreement, supporting their role as the reference standard when routine imaging yields equivocal results.

Compared with prior literature, our study differs in both methodological approach and population characteristics. While earlier studies have generally reported global, patient-based diagnostic performance of individual sequences [7,14,15], we performed a segment-based evaluation allowing a more detailed assessment in anatomically distinct venous structures, as diagnostic challenges in DVST are often localized rather than diffuse. Our results are broadly consistent with previous reports demonstrating the utility of diffusion- and susceptibility-based imaging [9,16], while extending these findings by showing how performance varies across individual sinuses. This sinus-specific perspective adds nuance to the existing literature and emphasizes that diagnostic pitfalls are not evenly distributed.

The demographic profile of our cohort differs from large Western series such as the ISCVT [17], which have consistently reported a female predominance largely attributed to hormone-related risk factors [17,18,19]. In contrast, our population demonstrated a male predominance, likely reflecting regional variations in underlying etiologies. In our cohort, non-hormonal factors—including infectious processes, inflammatory conditions such as Behçet’s disease, and hematologic disorders—accounted for a substantial proportion of cases, whereas hormone-related risk factors were relatively infrequent [15,20,21,22]. A subset of cases remained idiopathic. Although the mean age was consistent with the young adult onset typically described in DVST [15,22,23], the distribution of predisposing conditions differed from that of hormonally driven Western populations [24]. These differences are clinically relevant, as they may influence thrombus age at the time of imaging and consequently affect signal characteristics across MRI sequences, underscoring the importance of region-specific validation rather than direct extrapolation from landmark datasets.

Our findings further support the growing evidence that routine MRI can serve as a reliable initial screening tool for DVST without the need for contrast-enhanced imaging. Diffusion-weighted imaging demonstrated the strongest overall diagnostic contribution, consistent with prior studies emphasizing its high specificity when intraluminal hyperintensity is present [15,25]; however, as not all thrombi demonstrate restricted diffusion [26], interpretation should be integrated with complementary sequences. SWI provided additional value, given that clot composition produces a conspicuous blooming artifact, offering more objective interpretation than conventional flow-void assessment [23]. Conventional T1- and T2-WI, while useful, showed greater variability due to flow-related artifacts and dependence on thrombus age [27,28,29], findings in line with prior reports [9,16] and consistent with the complementary role of diffusion and susceptibility imaging.

**Figure 3:**
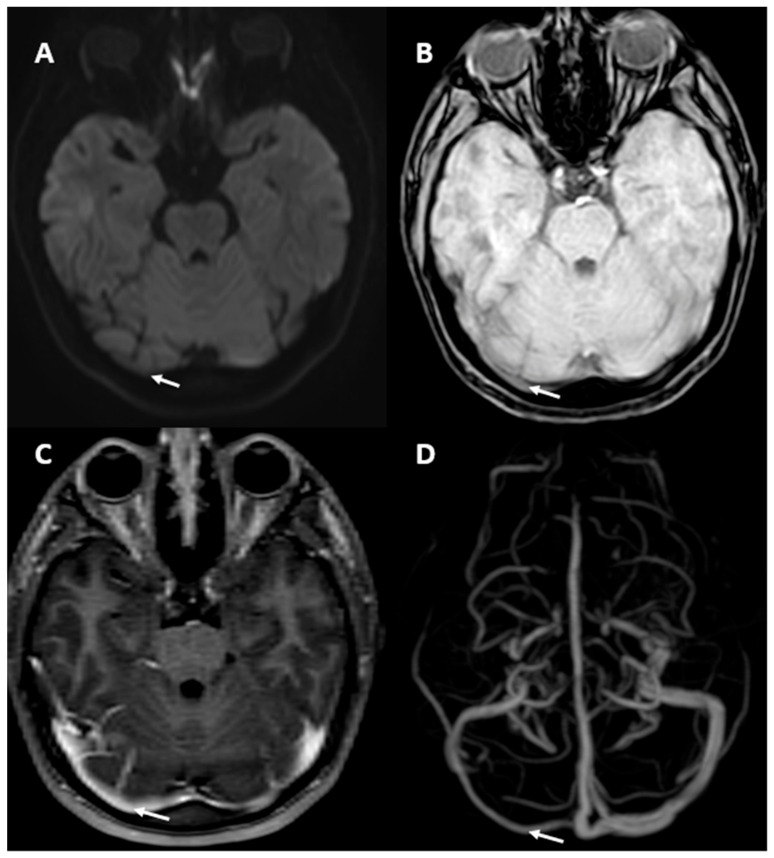
MR imaging findings of right transverse sinus hypoplasia in a representative case, shown in direct comparison with the thrombosis-specific signals demonstrated in Figure 2. The right transverse sinus is indicated by thick white arrows in all images. (**A**) DWI demonstrates no intraluminal restricted diffusion. (**B**) SWI shows no susceptibility-related blooming artifact. (**C**) CE-3D T1WI reveals no intraluminal filling defect. (**D**) MIP-CE-MRV confirms absence of filling defect with preserved sinus patency. The absence of thrombosis-specific MRI findings across all sequences is consistent with congenital hypoplasia rather than thrombotic occlusion, in contrast to the positive DWI (Figure 2E) and SWI (Figure 2F) findings observed in the thrombosis case.

A key contribution of our study is the demonstration that diagnostic performance is highly sinus-dependent [18,23,30,31]. The SSS was best evaluated using sagittal T1WI (along with SWI), reducing partial volume effects inherent to axial acquisitions. The transverse sinus was more reliably assessed using DWI- and SWI-based approaches, where slow flow and anatomical constraints may limit conventional sequences [23]. The transverse sinus remained the most challenging segment, prone to false-positive interpretations due to anatomical variants such as hypoplasia and flow asymmetry [14,30,32], with lower inter-reader agreement reflecting the difficulty of distinguishing slow flow from true thrombosis. A relevant diagnostic challenge in transverse sinus evaluation is the differentiation between true thrombosis and anatomical hypoplasia or aplasia [32,33]. Hypoplastic transverse sinuses typically demonstrate reduced caliber without associated intraluminal diffusion restriction on DWI or marked susceptibility-related blooming on SWI [30]. In contrast, venous sinus thrombosis more commonly shows intraluminal diffusion restriction and hypointensity with blooming artifacts on SWI, reflecting thrombus composition [26,27]. Representative imaging demonstrating this contrast between hypoplasia and thrombosis across DWI, SWI, and CE-MRV is provided in Figure 2 and Figure 3. However, overlap in imaging appearances may occur, particularly in chronic or partially recanalized thrombi [26,29]. Therefore, combined interpretation of DWI and SWI, together with assessment of contralateral sinus caliber and correlation with CE-MRV, is essential in equivocal cases [30,32]. The sigmoid sinus required careful integration of multiple sequences to overcome adjacent bone artifacts, with FLAIR demonstrating particularly strong inter-reader reproducibility. These findings reinforce that sequence selection should be adapted to the anatomical characteristics of each venous segment. Inter-observer agreement was generally high, particularly for diffusion- and susceptibility-based imaging, whereas conventional sequences demonstrated greater variability.

Diagnostic pitfalls remain an important consideration. False-negative findings may occur in the acute phase when thrombi are isointense on conventional sequences [27], whereas false positives are frequently related to anatomical variants or slow flow [14,30,32], particularly in the transverse sinus. Isolated findings on a single sequence should be interpreted with caution and corroborated with complementary techniques. In cases of persistent clinical suspicion or equivocal findings, CE-MRV and CE-3D T1WI remain essential [7,33,34,35].

This targeted multisequence approach allows efficient use of routine MRI while reserving contrast-enhanced techniques for inconclusive cases [7,35], helping reduce diagnostic delays and optimize resource utilization.

Non-contrast MR venography techniques, including time-of-flight MR venography (TOF-MRV) and phase-contrast MR venography, have gained renewed interest as contrast-free alternatives for cerebral venous evaluation. Although these techniques were not included in our imaging protocol, they may complement routine MRI assessment without requiring gadolinium-based contrast administration, particularly in patients with contraindications to contrast agents. Future studies incorporating non-contrast MRV techniques alongside routine MRI sequences may further refine diagnostic strategies for DVST within contemporary neurovascular imaging practice [3].

This study has several limitations. Firstly, the male predominance in our thrombosis cohort and the differences in sex distribution may limit direct comparability and generalizability with female-predominant Western cohorts. Secondly, the single-center retrospective design and requirement for a complete MRI protocol, including CE-MRV and CE-3D T1WI, may have introduced selection bias, potentially limiting generalizability to centers where contrast administration is not routinely performed. Thirdly, the limited number of thrombosis cases in certain segments, particularly the straight sinus, resulted in quasi-complete separation and statistical instability in logistic regression models, limiting the interpretability of individual sequence contributions in these subgroups. In particular, the observed 100% diagnostic accuracy for the straight sinus should be regarded as a preliminary finding, as it was derived from only five thrombosis-positive cases; this result warrants confirmation in larger, dedicated cohorts before definitive clinical conclusions can be drawn. Fourthly, our analysis did not stratify diagnostic performance by thrombus age (acute, subacute, or chronic), which is known to substantially influence signal characteristics on conventional sequences and may alter thrombus visibility. Acute thrombi may show variable signal intensity on T1WI (iso- to mildly hyperintense) and typically demonstrate hyperintensity on DWI due to restricted diffusion. Subacute thrombi are characterized by T1 shortening related to intracellular methemoglobin, whereas chronic thrombi may show partial recanalization and variable restoration of flow void with persistent organized thrombus. The limited number of cases precluded meaningful subgroup analysis; future studies with larger cohorts should incorporate temporal stratification to further refine sinus-specific diagnostic recommendations [5,29]. Fifthly, isolated cortical vein thrombosis was not evaluated, limiting applicability to dural sinus thrombosis specifically. Sixthly, MRI examinations were performed using both 1.5 T and 3.0 T scanners, with a similar distribution between groups; however, field-strength-related variability cannot be entirely excluded. In addition, heterogeneity in MRI acquisition protocols across vendors may have introduced further variability, as pre-contrast T1-WI was performed using either TSE or TFE depending on the system, and SWI was acquired using either single-echo or multi-echo techniques. These differences may have affected tissue contrast and susceptibility sensitivity, potentially introducing minor inconsistencies in sequence-based diagnostic performance. Seventhly, all examinations were contrast-enhanced, utilizing various gadolinium-based contrast agents. Although the route and protocol of administration were standardized, differences in contrast agent properties may have introduced minor variability in imaging characteristics. Eighthly, the DVST-enriched case–control design may have introduced spectrum bias, potentially overestimating diagnostic accuracy compared with unselected clinical populations. Finally, lack of routine digital subtraction angiography may have limited the detection of subtle venous abnormalities.

## 5. Conclusions

Routine brain MRI sequences are effective initial tools for evaluating DVST, providing high specificity overall; however, sensitivity varies across anatomical segments and sequences. Based on the findings, a practical stepwise diagnostic approach may be proposed for routine clinical practice. In patients with suspected DVST on initial non-contrast MRI, DWI and FLAIR can be used as primary sequences in global evaluation. For sinus-specific assessment, sagittal T1WI combined with SWI may be particularly useful for the superior sagittal sinus, DWI combined with SWI for the transverse sinus, DWI combined with FLAIR and T2WI for the sigmoid sinus, and SWI combined with axial T1WI for the straight sinus. When findings are equivocal or discordant across sequences, CE-MRV and CE-3D T1WI should be used as problem-solving techniques. This proposed hierarchical approach may improve diagnostic confidence while optimizing the use of contrast-enhanced imaging, but requires prospective validation before routine clinical implementation.

## Figures and Tables

**Table 1:** Representative imaging parameters.

Sequences	1.5 Tesla Fuji						1.5 Tesla Philips						3.0 Tesla Philips					
	TR (ms)	TE (ms)	Δ TE (ms)	Acquisition Matrix	TI	Flip Angle	TR (ms)	TE (ms)	Δ TE (ms)	Acquisition Matrix	TI	Flip Angle	TR (ms)	TE (ms)	Δ TE (ms)	Acquisition Matrix	TI	Flip Angle
Sagittal T1WI	489	11.1	N/A	256 × 180	N/A	90°	7.3	3.5	N/A	256 × 238	N/A	8°	7.8	3.8	N/A	252 × 240	N/A	8°
Axial T1WI	489	11.1	N/A	256 × 180	N/A	90°	450	15	N/A	230 × 188	N/A	90°	7.8	3.8	N/A	220 × 140	N/A	8°
Axial T2WI	5079	95	N/A	320 × 224	N/A	90°	7246	110	N/A	200 × 184	N/A	90°	3000	80	N/A	272 × 194	N/A	90°
FLAIR **	9000	117	N/A	256 × 200	2300	90°	4800	325	N/A	232 × 199	1660	90°	4800	304	N/A	228 × 217	1650	90°
DWI (b = 1000 s/mm^2^)	4221	88	N/A	120 × 148	N/A	90°	3056	75	N/A	152 × 94	N/A	90°	3482	86	N/A	152 × 106	N/A	90°
SWI	80	40	N/A	452 × 260	N/A	23°	52	12 *	11	272 × 184	N/A	20°	31	7.2 *	6.2	288 × 159	N/A	17°
CE-MRV	15.8	4.8	N/A	192 × 120	N/A	17°	12	8	N/A	352 × 352	N/A	10°	12	7.6	N/A	256 × 178	N/A	10°
CE-3D T1WI	10.7	6	N/A	256 × 200	N/A	12°	7.3	3.5	N/A	256 × 238	N/A	8°	7.8	3.8	N/A	252 × 240	N/A	8°

* On Philips systems (1.5 T and 3 T), SWI was acquired using a multi-echo protocol; TE indicates the first echo time (TE1), with subsequent echoes acquired at intervals defined by ΔTE. The Fuji 1.5 T system employed a single-echo gradient echo protocol. ** FLAIR sequences were acquired as 3D FLAIR on Philips systems.

**Table 2:** Global sequence-based performance.

Sequence(s)	Accuracy	AUC	Sensitivity	Specificity	TP	FN	TN	FP
DWI b1000 + FLAIR	97.9%	0.997	91.2%	100%	31	3	106	0
Any conventional sequence positive (T1WI/T2WI/FLAIR/DWI/SWI)	93.6%	0.958	100%	91.5%	34	0	97	9

DWI, diffusion-weighted imaging; b1000, b-value 1000 s/mm^2^; FLAIR, fluid-attenuated inversion recovery; T1, T1-weighted imaging; T2, T2-weighted imaging; SWI, susceptibility-weighted imaging; AUC, area under the curve; TP, true positive; FN, false negative; TN, true negative; FP, false positive.

**Table 3:** Sinus-based performance.

Venous Sinus	Sequence(s)	Accuracy	AUC	Sensitivity	Specificity	TP	FN	TN	FP
Superior sagittal sinus	T1WI sagittal + SWI	99.3%	0.969	93.8%	100%	15	1	124	0
Transverse sinus	DWI b1000 + SWI	97.9%	0.999	100%	97.3%	27	0	110	3
Sigmoid sinus	DWI b1000 + FLAIR + T2WI	98.6%	0.980	92.6%	100%	25	2	113	0
Straight sinus	SWI + axial T1WI	100%	1.000	100%	100%	5	0	135	0

DWI, diffusion-weighted imaging; b1000, b-value 1000 s/mm^2^; SWI, susceptibility-weighted imaging; FLAIR, fluid-attenuated inversion recovery; T2, T2-weighted imaging; T1, T1-weighted imaging; TP, true positive; FN, false negative; TN, true negative; FP, false positive; AUC, area under the curve.

**Table 4:** Global sequence-based inter-rater reliability.

Sequence	Kappa	SE	95% CI Lower	95% CI Upper
T1WI sagittal	0.863	0.050	0.764	0.961
T1WI axial	0.898	0.045	0.810	0.986
T2WI axial	0.821	0.057	0.709	0.932
FLAIR axial	0.875	0.046	0.785	0.965
DWI b1000	0.890	0.048	0.796	0.984
SWI	0.895	0.046	0.805	0.985
Contrast-enhanced MRV	0.980	0.020	0.942	1.000
Contrast-enhanced-3D T1WI	0.980	0.020	0.942	1.000

SE, standard error; CI, confidence interval; T1, T1-weighted imaging; T2, T2-weighted imaging; FLAIR, fluid-attenuated inversion recovery; DWI, diffusion-weighted imaging; b1000, b-value 1000 s/mm^2^; SWI, susceptibility-weighted imaging; MRV, MR venography.

**Table 5:** Sinus-based inter-rater reliability.

Sinus	Sequence	Kappa	SE	95% CI Lower	95% CI Upper
Transverse	Sagittal T1WI	0.826	0.059	0.710	0.942
	Axial T1WI	0.832	0.061	0.712	0.953
	Axial T2WI	0.660	0.085	0.492	0.827
	FLAIR	0.808	0.061	0.689	0.927
	DWI b1000	0.801	0.072	0.660	0.942
	SWI	0.833	0.061	0.714	0.952
	CE-MRV	0.953	0.033	0.888	1.000
	CE-3D T1WI	0.953	0.033	0.888	1.000
Sigmoid	Sagittal T1WI	0.863	0.060	0.745	0.980
	Axial T1WI	0.863	0.060	0.745	0.980
	Axial T2WI	0.793	0.069	0.657	0.929
	FLAIR	0.887	0.049	0.791	0.984
	DWI b1000	0.826	0.069	0.691	0.960
	SWI	0.832	0.066	0.702	0.962
	CE-MRV	0.977	0.023	0.931	1.000
	CE-3D T1WI	0.953	0.033	0.888	1.000
SSS	Sagittal T1WI	0.818	0.088	0.645	0.991
	Axial T1WI	0.858	0.080	0.701	1.000
	Axial T2WI	0.909	0.064	0.784	1.000
	FLAIR	0.868	0.074	0.722	1.000
	DWI b1000	0.743	0.110	0.528	0.958
	SWI	0.909	0.064	0.784	1.000
	CE-MRV	0.964	0.036	0.893	1.000
	CE-3D T1WI	0.964	0.036	0.893	1.000
Straight	Sagittal T1WI	1.000	0.000	1.000	1.000
	Axial T1WI	0.495	0.306	−0.105	1.000
	Axial T2WI *^a^	0.000	0.000	NC	NC
	FLAIR	1.000	0.000	1.000	1.000
	DWI b1000	1.000	0.000	1.000	1.000
	SWI	0.854	0.144	0.571	1.000
	CE-MRV	1.000	0.000	1.000	1.000
	CE-3D T1WI	1.000	0.000	1.000	1.000

*^a^ Statistical instability caused by zero cell counts (quasi-complete separation) resulted in an invalid confidence bound for this variable. SE, standard error; CI, confidence interval; SSS, superior sagittal sinus; FLAIR, fluid-attenuated inversion recovery; DWI, diffusion-weighted imaging; SWI, susceptibility-weighted imaging; CE-MRV, contrast-enhanced MR venography; CE-3D T1WI, contrast-enhanced three-dimensional T1-weighted imaging; NC, not calculable.

## Data Availability

Data are available from the corresponding author upon reasonable request.

## Abbreviations

DVST	Dural Venous Sinus Thrombosis
CE	Contrast-Enhanced
MRI	Magnetic Resonance Imaging
MRV	Magnetic Resonance Venography
SWI	Susceptibility-Weighted Imaging
DWI	Diffusion-Weighted Imaging
FLAIR	Fluid-Attenuated Inversion Recovery
SSS	Superior Sagittal Sinus
TFE	Turbo Field Echo
TSE	Turbo Spin Echo
mFFE	Multi-echo Fast Field Echo
